# Childhood stroke associated with protein C and S deficiency

**DOI:** 10.1111/cns.14479

**Published:** 2023-09-21

**Authors:** Hui Yu, Yao Zhang, Ting Wei, Wenqian Luo, Bin Liu

**Affiliations:** ^1^ Department of Neurology, Shandong Provincial Qianfoshan Hospital, School of Clinical Medicine Weifang Medical University Weifang China; ^2^ Department of Neurology The First Affiliated Hospital of Shandong First Medical University & Shandong Provincial Qianfoshan Hospital Jinan China; ^3^ Shandong Institute of Neuroimmunology Jinan China

## INFORMED CONSENT

The study was approved by the ethics committee of Shandong Provincial Qianfoshan Hospital (no. S405). We have obtained the patient's permission and informed consent for the publishing of her information and images.

Dear Editor,

Stroke is a serious threat to human health and life worldwide due to its high morbidity, disability, and mortality rates. Childhood stroke is an important cause of neurological disorders in children and most survivors suffer from severe physical complications impacting the remainder of their life. Protein C and S deficiency may contribute to the multifactorial etiology of stroke in early childhood. Here, we described a 10‐year‐old girl who presented with acute ischaemic stroke due to protein C and S deficiency.

A 10‐year‐old right‐handed girl was transferred from an outside hospital with right‐sided weakness for 11 h. She was the first child of his parents who were healthy. On examination, mental status, cranial nerves, reflexes, and sensation of the left upper and lower extremities were normal. Reflexes were also normal on the left side of the body. Right upper and lower extremities were flaccid, paralyzed, and areflexic and a plantar response could not be obtained. The pain perception and tactile sensation were normal on the right side.

**FIGURE 1:**
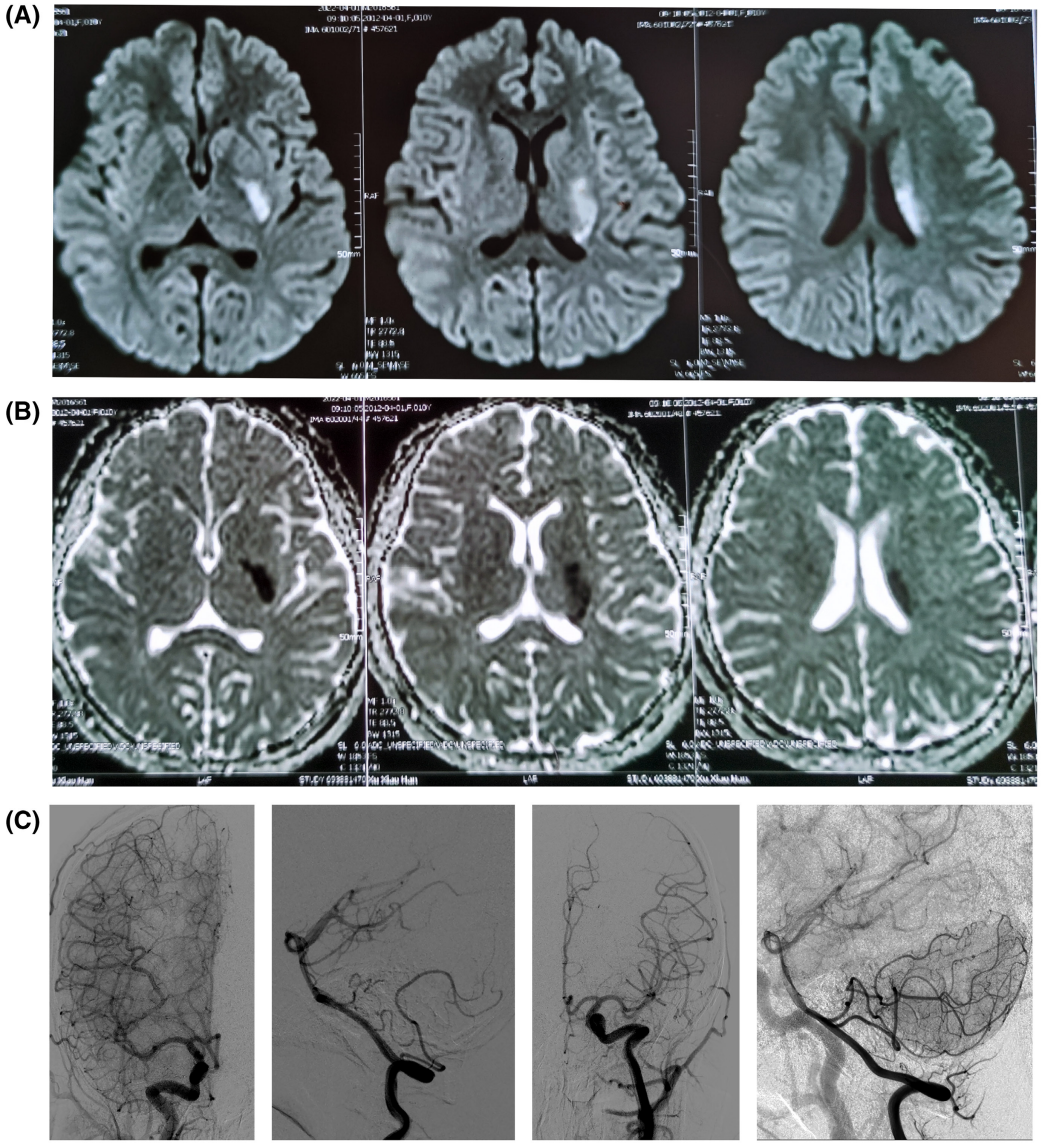
MRI and DSA images of this patient. Axial DWI (A) and ADC (B) images at the same level shows punctate foci of diffusion restriction in the left corona radiata and internal capsule. DSA show no vascular abnormality (C).

An urgent MRI of the brain was obtained 5 hours after presentation and showed diffusion restriction in the left corona radiata and internal capsule (Figure 1A,B). The extracranial carotid, vertebral, subclavian arteries, bilateral carotid, and vertebral systems, when evaluated by digital subtraction angiography, did not show any abnormalities such as plaque formation (Figure 1C). Cardiologic investigations including electrocardiogram, electrocardiographic (Holter) monitoring, and transesophageal (TE) echocardiography did not reveal any abnormality.

Laboratory tests: No abnormalities in routine blood parameters, coagulation routine, glucose, lipids, rheumatic immunity, infection markers, antiphospholipid antibodies, and ANCA. Deficiency of Protein C (37.6%, *N* = 70–140) and S (31.2%, *N* = 60–130) was finally diagnosed. This was confirmed on a repeat specimen 1 month later. She tested negative for PROC gene mutation.

The patient was treated with rivaroxaban (20 mg per day). She was discharged after taking rivaroxaban for 12 days without any adverse effects and continued taking rivaroxaban out of the hospital. We followed up the patient monthly for 18 months, and no symptoms of thrombosis occurred.

## DISCUSSION

Stroke is the leading cause of death in China. According to the Global Burden of Disease (GBD) 2019 report, the annual number of strokes and deaths due to stroke increased substantially from 1990 to 2019.^1^ The disease burden of stroke in China is increasing due to the aging population and the rising prevalence of stroke‐related risk factors. Additionally, stroke is now occurring at a younger age.^2^, ^3^ Childhood stroke is emerging as a serious and recurrent disorder, but studies remain largely descriptive. Because a stroke is not expected in young children, the diagnosis may be delayed.^4^ The causes of childhood stroke are distinct from those in adults. Identifying the etiology of childhood ischemic stroke helps prevent stroke recurrence. The major risk factors in children included congenital or acquired cardiac disease, aortic/cervical arteriopathy, bilateral cerebral arteriopathy (Moyamoya), small vessel arteriopathy, focal cerebral arteriopathy, severe thrombophilia such as in antiphospholipid antibody (APLA) syndrome and other proinflammatory disorders, genetic vasculopathy, and multiple infectious illnesses.^5^ Among the multiple causes of stroke in children, genetic defects in the coagulation system are increasingly being recognized.^6^ Protein C (PC) deficiency is a rare cause of the development of arterial thrombosis and ischaemic stroke. PC is a vitamin K‐dependent serine protease. Activated protein C inactivates activated factor V and factor VIII, thereby inhibiting thrombin production.^7^ Protein S (PS) enhances this process, and protein S is a cofactor for protein C. PC and PS deficiency disturbs the balance between procoagulant and anticoagulant proteins, and is thus responsible for thrombosis. The etiology of PC deficiency may be genetic or acquired. Hereditary PC deficiency occurs due to a mutation in the PROC gene. In this patient, genetic sequencing did not show abnormalities, so hereditary protein C deficiency was ruled out. Acquired protein C deficiency is mainly due to increased consumption or reduced production, including disseminated intravascular coagulation, recent thrombosis, hemolytic uremic syndrome, thrombotic thrombocytopenic purpura, oral anticoagulant protein C, vitamin K deficiency and liver disease.^8^ The association between PC deficiency and ischemic cerebral vessel disease is not yet well established.^9^ In one study, the authors indicated that PC and PS deficiency were not associated with stroke in adolescents.^10^ Yet, anecdotal cases have demonstrated a link between PC deficiency and ischemic stroke. Hence, eminent data are still required to confirm this relationship. The incidence of ischaemic stroke was lower than that of venous accidents in PC‐deficient patients. Treatment strategies for ischaemic stroke in children have been extrapolated from those used in adult cases. With regard to the treatment of PC deficiency, long‐term oral anticoagulation is thought to be beneficial in preventing the recurrence of arterial or venous thrombosis. Anticoagulants such as heparin, warfarin, aspirin, and clopidogrel are mainly used. We conducted a comprehensive search on PubMed and Google Scholar to identify case reports of protein C or protein S deficiencies associated with childhood stroke. The search was conducted from 1987 to 2023, in children from birth to 18 years of age, and the relevant articles were compiled and analyzed (Table 1). We found that aspirin and warfarin are the majority of choices in the treatment of stroke in children. There is no defined standard for treatment of Childhood stroke associated with PC and PS deficiency. In this case report, the patient was treated with rivaroxaban (a new oral anticoagulant) in the initial and long‐term prevention of thromboembolism, demonstrating the potential of rivaroxaban to treat thrombosis. The use of new oral anticoagulants provided new therapeutic options for stroke caused by PC or PS deficiency.

**TABLE 1:** Reported patients of childhood stroke associated with protein C or S deficiency.

References	Year	Age	Sex	Clinical features	PC level (%)	PS level (%)	Management
[11]	1993	26 months	Boy	Right hemiparesis	66	N/A	N/A
[11]	1993	6 years	Boy	Left hemiparesis	N/A	59	N/A
[12]	1993	22 months	Girl	Right‐sided weakness	36	N/A	Aspirin
[12]	1993	12 years	Girl	Left hemiparesis	54	13	Aspirin
[13]	1987	17 months	Girl	Left hemiparesis	44	N/A	Aspirin
[13]	1987	13 months	Boy	Right hemiparesis	72	38	Aspirin
[14]	1999	12 years	Girl	Left hemiparesis	60	N/A	Aspirin
[15]	1994	6 years	Boy	Dysarthria, right‐sided weakness	N/A	25	N/A
[16]	2004	5 years	Boy	Focal seizure	N/A	56	Warfarin
[17]	2002	3 years	Boy	Left hemiparesis	47	80	Anticoagulants were not used
[18]	2023	2.5 years	Girl	Left hemiplegia	28	N/A	Vitamin K antagonists
[19]	2005	3 years	Girl	Right hemiparesis	50	27	Warfarin
[20]	2001	15 years	Girl	Right hemiparesis	68	27	Anticoagulants were not used

Abbreviation: N/A, not available.

There is still a great deal of controversy about protein C and protein S deficiency as risk factors for stroke in children, and more studies are needed to validate this, as well as a large amount of clinical data still needed for the treatment of stroke in young people.

## FUNDING INFORMATION

This work was supported by the National Natural Science Foundation of China (grant no. 81601018) Natural Science Foundation of Shandong Province, China (ZR2021MH043), and the Academic promotion programme of Shandong First Medical University (2019QL013).

## CONFLICT OF INTEREST STATEMENT

The authors have declared that no competing interest exists.

## Data Availability

Data available on request from the authors.
